# Ultrafast Laser Material Damage Simulation—A New Look at an Old Problem

**DOI:** 10.3390/nano12081259

**Published:** 2022-04-08

**Authors:** Simin Zhang, Carmen Menoni, Vitaly Gruzdev, Enam Chowdhury

**Affiliations:** 1 Department of Material Science and Engineering, The Ohio State University, Columbus, OH 43210, USA; zhang.10584@osu.edu; 2 Electrical and Computer Engineering Department, Colorado State University, Fort Collins, CO 80523, USA; carmen.menoni@colostate.edu; 3 XUV Lasers Inc., Fort Collins, CO 80525, USA; 4 Department of Physics and Astronomy, University of New Mexico, Albuquerque, NM 87106, USA; vgruzdev@unm.edu; 5 Department of Physics, The Ohio State University, Columbus, OH 43210, USA; 6 Department of Electrical and Computer Engineering, The Ohio State University, Columbus, OH 43210, USA

**Keywords:** laser-plasma interaction, femtosecond laser, numerical, dielectric thin films

## Abstract

The chirped pulse amplification technique has enabled the generation of pulses of a few femtosecond duration with peak powers multi-Tera and Peta–Watt in the near infrared. Its implementation to realize even shorter pulse duration, higher energy, and higher repetition rate laser systems relies on overcoming the limitations imposed by laser damage of critical components. In particular, the laser damage of coatings in the amplifiers and in post-compression optics have become a bottleneck. The robustness of optical coatings is typically evaluated numerically through steady-state simulations of electric field enhancement in multilayer stacks. However, this approach cannot capture crucial characteristics of femtosecond laser induced damage (LID), as it only considers the geometry of the multilayer stack and the optical properties of the materials composing the stack. This approach neglects that in the interaction of an ultrashort pulse and the materials there is plasma generation and associated material modifications. Here, we present a numerical approach to estimate the LID threshold of dielectric multilayer coatings based on strong field electronic dynamics. In this dynamic scheme, the electric field propagation, photoionization, impact ionization, and electron heating are incorporated through a finite-difference time-domain algorithm. We applied our method to simulate the LID threshold of bulk fused silica, and of multilayer dielectric mirrors and gratings. The results are then compared with experimental measurements. The salient aspects of our model, such as the implementation of the Keldysh photoionization model, the impact ionization model, the electron collision model for ‘low’-temperature, dense plasma, and the LID threshold criterion for few-cycle pulses are discussed.

## 1. Introduction

The invention of chirped pulse amplification (CPA) method recognized with the Nobel Physics Prize awarded to Donna Strickland and Gerard Mourou in 2018 [1,2] has totally changed the rules of the game in the entire area of ultrafast lasers and ultrafast laser-material interactions. The high peak power of the CPA table-top ultrafast lasers is used to produce material modifications (e.g., laser-induced ablation [3], ultrafast melting [4], direct writing of waveguides [5], and periodic surface nano-structuring [6]) on a routine basis in many laboratories worldwide. Furthermore, the robustness of the architecture has allowed wide ranging industrial applications, e.g., eye and other surgery [7,8,9], medical device manufacturing [10] etc. Impressive advances in energy scaling and pulse duration reduction of CPA lasers have motivated the construction of large-scale ultrafast laser facilities capable of generating terawatt and petawatt output energy compressed into pulses as short as 100 fs [11,12]. Further increase of output power of the high peak-power ultrafast laser systems is one of the major trends in ultrafast laser science and technology. However, the progress of that development is substantially hindered by the laser-induced damage (LID) of optical materials used in critical laser components [13]. Significant research efforts are currently focused on studies of all aspects of high-power ultrafast interactions of femtosecond laser pulses with the transparent materials. These efforts are motivated by the need to better understand the LID mechanisms and improve the LID thresholds in order to unblock further progress in high-power ultrafast laser systems and their applications including inertial confinement fusion [11,12], compact particle accelerators [14,15,16,17] for various applications [18,19], novel neutron sources [20,21], material processing by laser ablation [22,23], surface nanostructuring [6,24,25,26], etc.

A cornerstone part of the research on LID improvement and applications of the ultrafast lasers for material processing is the study of ultrafast laser-dielectric interactions. In general, the interactions can be classified as: bulk of a dielectric (e.g., bulk LID or direct laser writing [5]), at the surface (e.g., surface nanostructuring [6,24] or ablation [3,27,28,29]), or in single or multiple layers (e.g., LID of optical multilayer interference coatings [30,31]). Experiments on ultrafast LID and ablation of dielectrics extensively reported over last two decades for all those three types of interactions have received a significant support by simulations. The most popular simulation approaches usually consider a one dimensional (1D) propagation for the laser-bulk and laser-surface interactions coupled to a module to describe the material response. The latter one includes generation of free carriers, energy transfer from a laser pulse to the material via nonlinear and free-carrier mechanisms of absorption, transient modifications of optical response by the free-carrier plasma, and modification of the material structure.

The free-carrier generation and nonlinear absorption are frequently simulated with the Keldysh photoionization model [32] that is believed to be valid in a broad range of laser intensity. It describes promotion of electrons from a valence band to a conduction band via simultaneous absorption of multiple laser photons at lower intensity (i.e., the multiphoton absorption) or via inter-band tunneling for higher intensity. Intense laser pulses can deliver energy to the laser-generated free carriers via inverse bremsstrahlung process (electron-photon-phonon interaction) that results in rapid thermalization of the free electrons, followed by the promotion of them to higher energy levels. The high-energy free electrons may become capable of transferring enough energy to the valence-band electrons via electron-electron collisions to promote the valence electrons to the conduction band. This collision-driven effect is referred to as impact or avalanche ionization and is usually characterized by extremely high ionization rates [33,34,35,36]. Simulations of this process employ various approaches [3,30,33,34,36,37,38] and are traditionally assumed to be some of key components of the modeling approaches for studies of LID. The pulses shorter than 200 fs usually prevent any immediate energy transfer from the excited electrons to the lattice during the laser pulse interaction with the solid. That energy transfer from the electrons to the lattice takes place after the exciting laser pulse has already left the solid (because the energy transfer time scale is longer than the pulse duration), which results in modification of the material structure, e.g., formation of laser-induced bulk gratings [5], transient defects [39], periodic structuring of surfaces [6] as well as ablation [3] and LID [34].

A majority of previous experiments and simulations of the ultrafast laser modification of material have been reported for the bulk and surface interactions. Although ultrafast laser interactions with multiple dielectric layers attract a very significant interest due to the fact that LID of such interference coatings is a major limiting factor for the enhancement of the output power of high-power laser systems [12], they are not as extensively studied [30,31,40,41,42]. The specific nature of the multilayer dielectric (MLD) coatings involves complicated propagation effects controlled by multiple reflections of the laser light from each interface and the resultant interference. The propagation results in the formation of local field maxima in the multilayers that can substantially affect the laser-dielectric interaction processes outlined above. A traditional approach to address the influence of the propagation effects is to employ some steady-state simulations of the field distribution in the multilayers. For example, Stuart et al., simulated the field enhancement ${\left|E\right|}^{2}$ in a HfO_2_/SiO_2_ MLD grating under the linear (i.e., intensity-independent) approximation and found that LID occurred at HfO_2_ pillars where ${\left|E\right|}^{2}$ is highest [33]. Neauport et al., found that LID threshold depends on ${\left|E\right|}^{2}$ by testing several similar SiO_2_/HfO_2_ MLD gratings, but they did not find damages at SiO_2_ ridges where ${\left|E\right|}^{2}$ is highest [43]. Although the multiple shot effects in S-on-1 damage tests could be a probable cause of damage to the layers before the pillar, it is more likely that the SiO_2_ pillars with a high bandgap withstood the high ${\left|E\right|}^{2}$ in contrast to their HfO_2_ counterparts in the layers. Thus, it is apparent that the ${\left|E\right|}^{2}$ criterion alone cannot capture the differences in laser resistance of different materials in MLD coatings. Using the same approach to simulate the field distribution, Gallais et al., studied the effects of nodular defects on LID threshold of MLDs in fs regime [44], and Chen et al., designed an asymmetric metal-dielectric grating in the hope to improve the LID threshold for fs pulses [45]. However, the ${\left|E\right|}^{2}$ distribution is still frequently employed for analysis of LID without properly addressing different laser resistance of materials. This happens because the steady-state modeling of field distribution in optical multilayers neglects modifications of optical properties of the layers due to free-carrier generation, formation of the free-carrier plasma and the resultant change in dielectric permittivities of the excited MLD layers. Since the ultrashort pulse propagation is dynamically coupled to the laser-induced modifications of the optical response, a dynamical approach modeling both the pulse propagation and the transient optical response of each dielectric layer is required to capture this interaction self-consistently.

In this connection we note that some important gaps and unresolved issues exist in the modeling of the nonlinear absorption and free-carrier generation. For example, evaluation of the photoionization rate by the Keldysh model of Ref. [32] requires the use of slowly-varying field amplitude. If dynamical modeling approaches, e.g., the finite-difference time-domain (FDTD) method, are employed, they deliver instant magnitude of the electric field of the laser-pulse at each time step for each space point. Being substituted into the Keldysh formula of Ref. [32], the instant field delivers an underestimated photoionization rate. In addition, original formulation of the Keldysh relations from Ref. [32] are frequently employed in the simulations [46,47,48,49,50] in spite of the well-known fact that that Ref. [32] contains several misprints [51]. Another significant issue is related to a proper formulation of the impact-ionization models. Stuart et al., proposed an approach based on the avalanche rate proportional to the laser intensity [34]. This simple model provided a good description of the ablation threshold versus the laser pulse duration down to some 10 ps. For fs pulses, the avalanche coefficients were obtained from fitting some experimental data [27]. Another branch of avalanche models takes the ionization energy into account [38,49,52,53]. However, these methods fail to take the electron energy absorption and distribution into account, leading to exaggeration of the ionization rate. In the presence of significant electron heating by the laser pulse, the impact ionization model proposed by Keldysh can address this problem [37]; however, under this approach, the effect of ponderomotive energy on the impact-ionization threshold remains unclear [36,54]. In our previous work, we proposed a two dimensional (2D) dynamic coupled model based on the FDTD algorithm and applied it in evaluation of LID thresholds of MLD mirrors and gratings. The simulation results agree well with the measurements [55,56,57]. In this paper, we summarize the improved simulation model that combines the FDTD propagation module with the material-response module and discuss several important aspects of implementing the dynamic coupled model for simulation of the ultrafast laser-matter interaction in MLD layers. Since the improved approach provides both an improved view of the fundamental mechanisms of the LID and a significant guidance for designing MLD systems for high-power ultrafast laser applications, this publication may be of high interest to several research communities.

The paper is organized as follows: Section 2 presents the FDTD algorithm that solves the pulse propagation and electronic dynamics, and the parameters used for the coating materials discussed here; Section 3, presents the benchmarking results using bulk fused silica and discusses the validity of energy and critical-free-carrier-plasma-density criteria of LID threshold, which, then is applied to a MLD mirror and a MLD grating system; finally, Section 4 deals with (1) the error arising from the misprint in the Keldysh formulae, (2) the effect of the ponderomotive energy on the impact ionization, and (3) the error introduced by applying the instantaneous electric field strength in the Keldysh formulae. We also broach the issues with implementation of the Keldysh photoionization theory in case of few-cycle laser pulses that are produced by the monochromatic approximation of that model.

## 2. Materials and Methods

### 2.1. Coating Designs and Materials Properties

**Figure 1:**
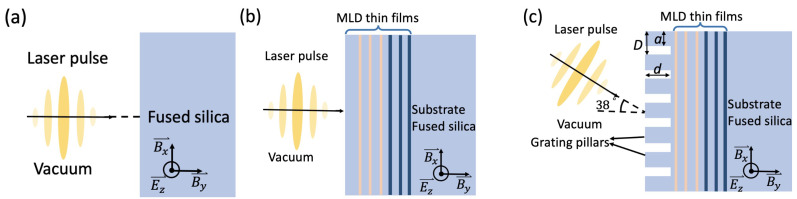
Schematics of simulation model. (**a**) Bulk fused silica; (**b**) MLD mirror; (**c**) MLD grating. *D* is the grating period. *a*/*D* is the duty cycle. *d* is the etch depth.

We first benchmarked our model by comparing the simulation results with laser damage test measurements on fused silica. In order to compare to the measurements in Ref. [47], we choose a laser pulse with a center wavelength λ = 800 nm and a full width of half maximum (FWHM) of intensity profile from 7 fs to 100 fs at normal incidence for the simulation (see Figure 1a). The temporal intensity profile is sine^2^, and the focal spot is Gaussian. The choice of the temporal profile is a standard practice to avoid ripples in the spectral domain.

Then, we evaluated the LID thresholds of a MLD mirror and a MLD grating using this model. The MLD mirror design consists of 40 intercalating SiO_2_ and HfO_2_ layers designed for λ = 800 ± 50 nm and normal incidence (see Figure 1b). The theoretical reflectance is 99% in the working wavelength range, 800 nm ± 50 nm, and its maximum reflectance is 99.5%. The MLD grating design (period = 572 nm, duty cycle = 42%, etch depth = 1.02 μm) consists of 48 alternating SiO_2_, HfO_2_, and Ta_2_O_5_ layers. It is designed for λ = 800 nm, *s* polarization, and 38° angle of incidence (AOI) (see Figure 1c). The theoretical diffraction efficiency of the grating is >90% for wavelengths from 785 nm to 845 nm, and its maximum diffraction efficiency is 97%.

In the simulations for the mirror and the grating, the center wavelength of the laser pulse is 800 nm. The pulse is *s*-polarized, and its FWHM duration is 50 fs. The material parameters used in the simulations are listed in Table 1 [57]. *n* is the refractive index at λ = 800 nm; $m^{*}=1/\left(1/m_{e}^{*}+1/m_{h}^{*}\right)$ is the reduced effective mass, in which $m_{e}^{*}$, and $m_{h}^{*}$ are the effective electron and hole masses, respectively. $m_{e}$ is the free electron mass. The model contains recombination time $\tau_{\mathrm{recomb}}$ as a phenomenological parameter to characterize free-carrier relaxation without referring to a specific mechanism. It can be extracted from experimental data [58].

### 2.2. Core Algorithm

The core of the FDTD algorithm consists of three modules: (a) a propagation module that solves Maxwell’s equations for the electro-magnetic field; (b) an ionization evaluator that evaluates the ionization rate, total density of laser-generated free carriers, and energy distribution of the conduction-band electrons; and (c) a transient-optical-response evaluator that calculates modifications of refraction and absorption from laser-driven currents of the laser-generated free-carrier plasma. The propagation module is described in Section 2.2.1 in detail. The implementation of the Keldysh photoionization model and impact ionization is explained in Section 2.2.2. Section 2.2.3 explains our approach to evaluation of the transient optical response based on the electron collision model for dense plasma at low temperatures.

#### 2.2.1. FDTD Algorithm

The basic structure and functioning of the propagation module are explained on the basis of 1D propagation model to avoid unnecessary details and complications. The approach utilized to digitize Maxwell’s equations for the 1D propagation geometry can be easily generalized to 2D and 3D cases. For ease of reading, the nomenclature of involved variables and quantities is summarized in Table 2.

The propagation module essentially employs the FDTD method [59] to numerically solve Maxwell’s equations. Following the traditional approach, we employ central differences to reduce the differential Maxwell’s equations to the finite-difference relations. Correspondingly, $J_{D}$, $n_{e}$, $T_{e}$ are calculated at the first half time step at each time iteration as

(1) $$n_{e}^{t+\frac{\Delta t}{2}}=n_{e}^{t-\frac{\Delta t}{2}}+\Delta tf\left(n_{e}^{t}\right)\omega_{\mathrm{ph}}^{t}+\Delta tf\left(n_{e}^{t}\right)n_{e}^{t}\omega_{\mathrm{col}}^{t}-\frac{\Delta tn_{e}^{t}}{\tau_{\mathrm{recomb}}},$$

(2) $$T_{e}^{t+\frac{\Delta t}{2}}=\left(\Delta tJ_{D}^{t}E^{t}-\Delta tW_{\mathrm{ion}}^{t}\omega_{\mathrm{col}}^{t}n_{e}^{t}f\left(n_{e}^{t}\right)+n_{e}^{t-\frac{\Delta t}{2}}qT_{e}^{t-\frac{\Delta t}{2}}\right)/\left(qn_{e}^{t+\frac{\Delta t}{2}}\right),$$

(3) $$J_{D}^{t+\frac{\Delta t}{2}}=J_{D}^{t-\frac{\Delta t}{2}}-\Delta t\beta^{t}J_{D}^{t}+\frac{q^{2}n_{e}^{t}}{m_{e}}\Delta tE^{t},$$

where *q* is the electron charge. The time step, Δt, and spatial step, Δz, must satisfy the Courant-Friedrichs–Lewy condition. $W_{\mathrm{ion}}$ is the impact ionization energy. The calculation of transient material parameters, β, $\omega_{\mathrm{ph}}$, and $\omega_{\mathrm{col}}$, is explained in Section 2.2.2 and Section 2.2.3. Comparing to the multiple-rate-equation model [60], Equation (1) is an engineering model for practical application that takes considerably less computational time. We also note that the proposed model neglects free-carrier diffusion. This is a reasonable approximation for the disordered dielectric materials we simulate since their disordered structure does not support any reasonable diffusion within the time intervals characteristic of the laser-material interactions discussed below.

A straightforward application of the photoionization and impact-ionization models frequently predicts substantial de-population of the valence band [47,54] that is referred to as total or near-total ionization. However, previous publications [61,62,63] suggest that promotion of about 10% of the valence-band electrons to the conduction band results in substantial modification of phonon spectrum and distortions of the energy bands. Therefore, it is reasonable to expect substantial changes of the electron dynamics and nonlinear absorption and even modifications of material structure [64,65] at the de-population of the valence band well below 100%. Since incorporation of those processes into the traditional models of ultrafast high-intensity laser-solid interactions focused on the laser-driven free-carrier generation is a challenge, those models neglect those processes. To address this issue, we incorporate a de-population factor into the rate Equation (1) given by the following equation:

(4) $$f\left(n_{e}^{t}\right)=1-\frac{n_{e}^{t}}{n_{0}-n_{e}^{t}},$$

where $n_{0}$ is the total valence-band population prior to the laser action. We assume $n_{0}$ is four times the amount of molecules since there are 4 Si-O bonds per molecule. This reasoning was also experimentally and computationally validated by our previous work on femtosecond LID of germanium over a range of laser wavelengths from near- to mid-IR [66]. Equation (4) suggests saturation of the free-electron density at the level of about 50% of the valence-band density. This criterion is supported by a statistical-mechanics reasoning that the rate of electron excitation is proportional to the probability of electron transitions and density of the electrons available for the transitions [67]. As long as the conduction-band electron density is much lower than the valence-band electron density, electron up transitions (i.e., valence-to-conduction-band) dominate over the down conduction-to-valence-band transitions. When population of the conduction band approaches 50% of that of the valence band, rate of the down transitions is expected to increase and become comparable to the rate of up transitions since both are driven by the same laser-pulse electric field and involve the same electron states in the same bands. With further increase of the free-electron density, the down transitions can be expected to dominate over the up transitions till the situation is driven to the equilibrium regime with nearly equal splitting of the total valence-band population between the two involved bands. This reasoning is reflected by the equation above.

It is also important to note that the process of valence-band de-population is frequently addressed via introduction of the following factor in Equation (1):

(5) $$f^{\prime }\left(n_{e}^{t}\right)=1-\frac{n_{e}^{t}}{n_{0}},$$

That factor reduces to zero with the conduction-band density approaching the initial valence-band electron density, i.e., in the case of nearly total ionization. However, the Keldysh photoionization theory [32] and the Keldysh impact ionization model [37] rely on the assumption that the total conduction-band population generated by those ionization processes is much smaller than the initial valence-band population. Therefore, Equation (5) cannot support the simulations in the regime of nearly total ionization when the ionization models are invalid. A majority of the previous simulations [47,54] neglect this fact by assuming the LID is initiated as soon as the conduction-band electron density reaches the critical-plasma density at laser wavelength that is about $10^{21}$ cm ${}^{-3}$ at near-infrared wavelengths, i.e., is about 2 orders of magnitude smaller than $n_{0}$. However, over-critical free-electron densities can be reached in the ultrafast laser-dielectric interactions [29,66], and the ionization processes should be treated more accurately.

The superscript *t* and the subscript *z* denote the current time point and the space coordinate, for which the electric and magnetic fields are evaluated. Following the FDTD method [59], electric field *E* and magnetic field *B* are evaluated at two different instants of time that stay apart by half-step. Correspondingly, Maxwell’s equations are written as

(6) $$B_{z+\frac{\Delta z}{2}}^{t+\frac{\Delta t}{2}}=B_{z+\frac{\Delta z}{2}}^{t-\frac{\Delta t}{2}}-\frac{\Delta t}{\Delta z}\left(E_{z+\Delta z}^{t}-E_{z}^{t}\right),$$

(7) $$E_{z}^{t+\Delta t}=E_{z}^{t}-\frac{c^{2}\Delta t}{\Delta z\epsilon_{r}}\left(B_{z+\frac{\Delta z}{2}}^{t+\frac{\Delta t}{2}}-B_{z-\frac{\Delta z}{2}}^{t+\frac{\Delta t}{2}}\right)-\frac{\Delta t}{\epsilon_{r}\epsilon_{0}}\left(J_{D}^{t+\frac{\Delta t}{2}}+J_{p}^{t+\frac{\Delta t}{2}}\right),$$

(8) $$J_{p}^{t+\frac{\Delta t}{2}}=\frac{\epsilon_{\mathrm{eff}}^{t}\omega_{\mathrm{ph}}^{t}\left(n_{0}-n_{e}^{t}\right)}{A_{\mathrm{cyc}}^{t}n_{0}}\frac{E^{t}}{|E^{t}|},$$

where $\epsilon_{r}=n^{2}$ and $\epsilon_{0}$ are the relative permittivity of the medium and the dielectric constant in the vacuum, respectively; *c* is the speed of light in vacuum. $A_{\mathrm{cyc}}^{t}$ is the amplitude of laser-pulse electric field evaluated over the nearest cycle.

Given $E_{z}^{t}$ from Equation (7), $J_{D}^{t}$, $n_{e}^{t}$, and $T_{e}^{t}$ are evaluated from Equations (1)–(3). One disadvantage of this leapfrog algorithm is that the strong nonlinear scaling of the photoionization rate with electric-field amplitude introduces an instability to the simulations. To partly suppress it, we evaluate the plasma current as follows:

(9) $$J_{D}^{t}=J_{D}^{t-\frac{\Delta t}{2}}-\frac{\Delta t}{2}\beta^{t}J_{D}^{t-\frac{\Delta t}{2}}+\frac{\Delta tq^{2}n_{e}^{t-\frac{\Delta t}{2}}}{2m_{e}}E^{t},$$

Equation (9) evaluates the current, free-carrier density, and electric field on the right hand side at the time steps Δt/4 instead of Δt/2. In fact, Equation (9) is a reformulation of the Drude model of free-carrier contribution to the optical response. Although Equation (9) is beyond the central-difference approach of the traditional FDTD method [59], it helps to suppress the instability. The perfect matched layers (PMLs) have been applied in this FDTD simulation to absorb the EM wave at the boundaries [59].

#### 2.2.2. Ionization

Ionization is pivotal in ultrafast laser-matter interaction because the electron-hole plasma generation can modify the materials’ refractive indices and affect the pulse propagation. In MLD coatings, the plasma can cause a shift in electric field enhancement. Direct absorption of photons with energy below the bandgap is impossible, except for the multiphoton and tunneling photoionization processes that depend nonlinearly on laser intensity, which can occur when the intensity is sufficiently high.

In our approach, we employ the Keldysh photoionization model originally reported in Ref. [32]. First, it has been extensively utilized to simulate the laser-induced generation of free carriers in a majority of publications. Second, in contrary to other approaches, the Keldysh model has been verified via comparison of the Keldysh formula with available experimental data on one-photon and two-photon absorption in semiconductors and dielectrics [68,69,70,71]. In particular, it shows perfect agreement with measured spectra of one-photon absorption of typical semiconductors (e.g., GaAs) near the fundamental absorption edge [68]. Theoretical analysis [69] also shows a minor (by about 20%) underestimation of one-photon absorption rate by the Keldysh formula as compared to the regular perturbation theory. For the two-photon absorption, the original Keldysh model [32] predicts the rates that are 1 to 2 orders of magnitude smaller than those measured in experiments [70,71]. The difference is explained by the fact that the original Keldysh formula [32] contains a misprint fixed in Ref. [72] and also neglects possible contributions from multiple valence bands [73] as well as degeneracy of the valence and conduction bands [68,71]. Estimations of the nonlinear-absorption rates by the Keldysh formula can be further improved if proper energy-momentum relations are utilized since the Keldysh approach demonstrates a significant influence of energy-momentum relation on the photo-ionization rate [74]. When all those factors are properly taken into account, the Keldysh formula delivers the magnitudes of nonlinear absorption that coincide with experimental data with accuracy of some 30–50%. That is an excellent result of a quantum-mechanical theory that employs no fitting parameters.

**Figure 2:**
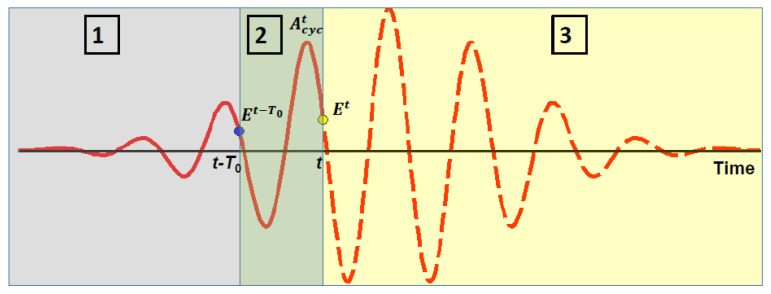
A sketch of time profile of electric field at a fixed space point. Time point *t* is the current point where electric field $E^{t}$ is evaluated from the FDTD equations. Areas 1 and 2 contain the time points where electric field has been evaluated by the FDTD method. Area 3 contains the “future” time points where electric field will be evaluated at later steps. Area 2 is the full cycle that is the nearest to the current time point *t*.

Although the Keldysh photoionization model [32] has been extensively utilized to evaluate the photoionization rate in modeling of ultrafast laser-solid interactions [47,48,57,75,76], an appropriate implementation of this model in the dynamic propagation simulations such as the FDTD method faces several fundamental challenges. First, the Keldysh model employs the approximation of given electric field of laser radiation, i.e., all parameters of the field (amplitude, phase, and frequency) are assumed to be known for an arbitrary time point at any space location in the interaction volume. This is not the case for the FDTD simulations since they deliver magnitudes of electric field only for moments of time that precede the time point, at which the field is currently evaluated for a given space location (Figure 2). This means, in particular, the peak magnitude of the laser-pulse electric field is not defined for the time points of the first half of a laser pulse prior to the pulse peak (see Figure 2). Second, the Keldysh model relations [32] are formulated in terms of cycle-averaged values, e.g., the effective band gap is evaluated via cycle-averaged energy of laser-driven electron and hole oscillations. Moreover, the photoionization rate of the Keldysh model is also averaged over a cycle according to the Keldysh calculation procedure [32]. Evaluation of those cycle-averaged parameters from the data delivered by the FDTD simulations at each space point requires storing electric-filed values for all time moments within a one-cycle interval that immediately precedes the current time point (see Figure 2). Those field values are stored as a vector for each space point, are updated at each time step of the FDTD simulations, and are utilized to evaluate amplitude of electric-field variations in time within that cycle interval. That amplitude is substituted into the Keldysh photoionization model to evaluate the instant photoionization rate. As we discuss below in Section 4, this approach provides larger magnitude of the photoionization rate compared to those obtained from substitution of instant electric-field magnitude into the Keldysh formula.

Third, the cycle-averaged approach for modeling of the photoionization assumes slow variations of pulse-envelope compared to field oscillations at the center frequency of laser-pulse spectrum [51,77]. With reduction of pulse duration towards few optical cycle, this requirement may be violated, and some corrections to the Keldysh-type model are required [77,78]. However, implementation of those corrections into the FDTD simulation meets extra challenges since they depend on carrier-envelope phase that is well defined within one cycle around the moment of peaking laser-pulse electric field [78]. Correspondingly, that parameter cannot be evaluated by the FDTD approach at the time moments prior to the laser-pulse peak. Implementation of this specific correction to the photoionization model for the FDTD simulations requires a separate and very accurate treatment to be discussed elsewhere. For this reason, we use the Keldysh photoionization model in the FDTD simulations reported in this paper for pulses as short as 7 fs in the near-IR regime (but no shorter), for which the corrections are estimated to be small [41,77].

Finally, the direct implementation of the Keldysh model via evaluation of the photoionization rate from the analytical formulations in Ref. [32] represents a significant challenge since the evaluation procedure requires time-consuming calculation of several special mathematical functions. Moreover, the slow amplitude of the Keldysh-type photoionization rate contains a sum that is evaluated via a few terms only in the multiphoton regime, i.e., at low intensity. With increase of laser intensity and transition to the tunneling regime, the number of the terms to be evaluated in the slow-amplitude sum grows as $\gamma^{-2}$ with the Keldysh adiabatic parameter [32] and can vary from few hundreds to few tens of thousands at the laser intensity characteristic of LID and ablation thresholds. Performing such evaluations at each space-time step substantially (by 1–2 orders of magnitude) slows down the entire dynamical simulation by the FDTD method. Some simulations approach this challenge by use of simpler analytical relations for the multiphoton (low-intensity) and tunneling (high-intensity) regimes [36,79] with some interpolation between them. However, such approaches introduce significant errors for the transition ionization mode between the multiphoton and tunneling regimes. For the dynamic simulations, the errors may result in unpredictable instability. To properly address this computational challenge without substantial increase of computational costs, the photoionization rate was calculated from the Keldysh model from given laser-pulse and material parameters in advance, was tabulated as function of peak intensity for reasonable steps of intensity variation, and was then utilized with accurate interpolation between the tabulated values.

#### 2.2.3. Electron Collision

Another ionization mechanism relevant to the ultrafast laser damage process is the impact ionization. Here we apply Keldysh’s model, which can be written as [37]

(10) $$\omega_{\mathrm{col}}=\int_{0}^{\infty }\omega_{0}\left(W\right)f_{e}\left(W\right)dW,$$

(11) $$\omega_{0}\left(W\right)=\left\{\begin{array}{cc} \alpha_{0}{\left(\frac{W}{W_{\mathrm{ion}}}-1\right)}^{2}, & \mathrm{if}\;\;W\geq W_{\mathrm{ion}},\\ 0, & \mathrm{else}, \end{array}\right.$$

where $f_{e}\left(W\right)$ is the electron energy distribution. In the present work, we assume it to be Maxwellian, although we understand that this assumption neglects the non-thermal/ballistic nature of the laser driven free carrier population, which will affect the impact ionization process. The addition of such kinetic aspects of impact ionization to our model and how it affects the whole laser damage process will be presented in a future work. Among the other parameters, $\alpha_{0}$ is a constant that depends on material parameters, taken to be 1.5 fs^−1^ for fused silica [54]. We also assume the same value for hafnia and tantala in this study because of the lack of availability of measured values for these materials. The ionization energy that fulfills the energy and momentum conservation is $W_{\mathrm{ion}}=\frac{1+2\mu }{1+\mu }\epsilon_{\mathrm{eff}}$ with $\mu =m_{e}^{*}/m_{h}^{*}$ [36,80] and $\epsilon_{\mathrm{eff}}$ to be the cycle-averaged effective band gap evaluated from the Keldysh photoionization model. Equation (11) implies that only electrons with energy surpassing $W_{\mathrm{ion}}$ can contribute to the impact ionization.

The electron collision frequency is a critical parameter in simulating the varying conductivity of plasma and the resultant absorption. The Spitzer scattering theory is usually used to describe the electron-ion collision frequency, which is written as [81]

(12) $$\beta_{\mathrm{sp}}\;\left[s^{-1}\right]=2.91\times 10^{-6}Z_{i}n_{e}\;\left[{\mathrm{cm}}^{-3}\right]\;\ln\Lambda \left(n_{e},T_{e}\right)T_{e}^{-3/2}\;\left[\mathrm{eV}\right],$$

where $Z_{i}$ is the ionization state of the nuclei; lnΛ is the Coulomb logarithm. Note that $\beta_{\mathrm{sp}}$ assumes short-range interactions of high-speed charged particles in a dilute plasma.

However, when the electron temperature is as low as a few eV, lnΛ becomes negative for the solid-density plasma, which means the electrons experience significant angular deviations and the assumption of Equation (12) becomes invalid. As the impact parameter *b* is less than the classical minimal impact parameter, $b_{\min}^{\mathrm{cl}}$, the classical “hard” collision becomes dominant and can be written as [82]

(13) $$\beta_{\mathrm{hard}}≅2\pi n_{i}v{\left(b_{\min}^{\mathrm{cl}}\right)}^{2},$$

(14) $$b_{\min}^{\mathrm{cl}}\left[m\right]≅4.8\times 10^{-10}\frac{Z_{i}}{T_{e}\;\left[\mathrm{eV}\right]},$$

where *v* is the electron velocity; $n_{i}$ is the ion density. $\beta_{\mathrm{hard}}$ is generally neglected because it is smaller than $\beta_{\mathrm{sp}}$ by a factor of 1/(2lnΛ) when the net Coulomb collision frictional force dominates (i.e., lnΛ≫1). In this case, collision frequency should consider both the cumulative small-angle and large-angle collision and be written as

(15) $$\beta =\left\{\begin{array}{cc} \beta_{\mathrm{sp}}+\beta_{\mathrm{en}}+\beta_{\mathrm{hard}}, & \mathrm{if}\;\;\ln\Lambda >0,\\ \beta_{\mathrm{en}}+\beta_{\mathrm{hard}}, & \mathrm{else}, \end{array}\right.$$

where the electron-neutral collision frequency $\beta_{\mathrm{en}}=2\times 10^{-7}n_{n}\;\left[{\mathrm{cm}}^{-3}\right]\;T_{e}^{1/2}\;\left[\mathrm{eV}\right]$ [54], in which $n_{n}$ is the neutral density.

We note that the recombination of free electrons is described by a phenomenological relaxation-time approach since measured relaxation time is frequently the only relevant parameter available from experimental data [58]. We also neglect the Auger recombination, since the strong reduction of its rate with the increase of band gap [83] suggests that this process is not significant for the wide-band-gap dielectrics under consideration. Similar treatment can be found in the simulations by the PIC methods, but the “hard” collision component is introduced in the scattering parameter, and the regimes for different collision mechanisms are separated by a threshold velocity [84]. Nevertheless, Equation (15) does not take the electron-phonon or the electron-defect collisions into consideration [85]. Electron conductivity is affected by any collisions that change the electron momentum. While the electron-electron collisions lead to a mutual exchange of momenta between the two colliding free electrons and do not change the entire sub-system of free electrons and the conductivity, the electron-phonon and electron-defect collisions can affect the electron conductivity. However, modeling of these processes in amorphous materials is challenging and not supported by sufficient experimental data. Therefore, these two processes are not modeled in this work to avoid the use of any nonphysical fitting parameters.

## 3. Results

In Section 3.1, we simulated the interaction of femtosecond laser pulses and fused silica, evaluated LID thresholds based on the energy density and critical electron density criteria, and compared obtained results with available experimental data on LID of fused silica by ultrashort laser pulses. In Section 3.2 and Section 3.3, we report simulations for a MLD mirror and a MLD grating and estimate their LID thresholds from the simulation results. The simulation parameters and MLD layer designs are introduced in Section 2.1. Note that the impact ionization is incorporated into our FDTD model with the ionization thresholds affected by the ponderomotive energy for all simulations in this work except those of Section 4.2.

### 3.1. Bulk Fused Silica

Fused silica is ubiquitous in ultra-intense laser systems, and there have been sufficient LID threshold measurements of bulk fused silica for different pulse durations [27,47]. Therefore, we chose it to benchmark our simulation model using the material parameters in Section 2.1 (see Figure 1a). In all simulations of bulk fused silica in this paper, the focal spot radius of the laser pulse is 23.6 μ m and the range of laser fluence is chosen to compare the simulation results to the measurements in [27,47]. It is important to note that changing the laser focal spot size in our simulations has negligible effect in determining LID threshold, and a particular value is chosen in consideration of physical validity and computational constraints [57].

**Figure 3:**
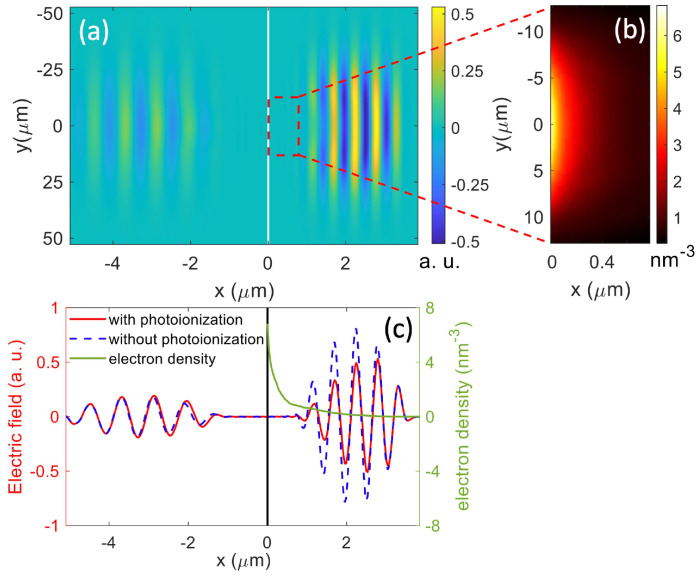
(**a**) Electric field distribution normalized to the amplitude of the incident pulse after the pulse leaves the target; (**b**) Electron density in the marked area; (**c**) Lineouts of electric fields (left panel) and electron density (right panel) with and without photoionization along *y* = 0. The vertical lines in (**a**,**c**) denote the air-and-target interface. The space on the left is air. The horizontal axes and scales in (**a**,**c**) directly correspond to each other.

Figure 3a shows that the pulse propagation becomes nonlinear at the trailing edge of a 7 fs pulse at peak fluence of 1.4 J/cm ${}^{2}$. The reflection slightly increases as the transmission decreases, and the wavefront shifts (also see Figure 3c). This is due to the plasma formation at the “hot” zone that causes the local modification of permittivity (see Figure 3b) [29]. As shown in Figure 3c, the dense plasma formed at the surface immediately renders the dielectric metallic and leads to a skin depth $l_{p}=\lambda /\left(4\pi \kappa \right)$ of 132 nm. κ is the imaginary part of the refractive index [47]. This short skin depth efficiently quenches the subsequent ionization further into the target. Consequently, the electron density falls to 1/e of its peak value at a depth of 224 nm.

**Figure 4:**
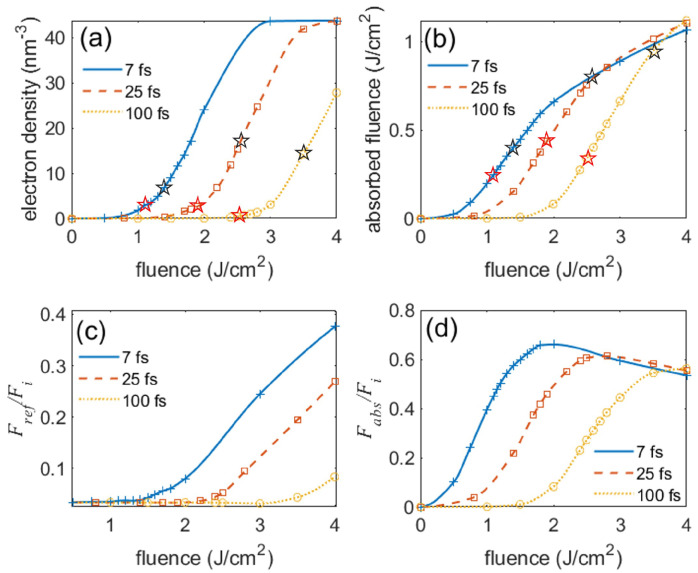
Simulation results for the interaction of bulk fused silica and pulses of various parameters along *y* = 0 (see Figure 3a). (**a**) Peak electron density; (**b**) Cumulative absorbed fluence; (**c**) Fractional reflected fluence; (**d**) Fractional absorbed fluence. The red and black stars represent the LID and ablation thresholds in [27,47], respectively. The crosses, squares, and circles represent the simulation data points for the 7 fs, 25 fs, and 100 fs pulses, respectively. The lines drawn through these points are merely to guide the eye.

On one hand, the plasma generation and pulse propagation have similar trends for different pulse durations. As the incident fluence rises, the electron density increases exponentially at low fluence and saturates at high fluence (see Figure 4a). Figure 4b,d show that the total and fractional cumulative absorbed fluences are correlated with the electron density. Once the electron density approaches a quarter of the total valance-band electrons available for ionization, $n_{0}/4$, the absorbed fluence starts to increase at a decreasing slope. Meanwhile, the fractional absorbed fluence declines, accompanied by the growth of fractional reflected fluence in Figure 4c. On the other hand, the pulse duration makes a significant difference near and above the LID thresholds. Figure 4a,b shows that, in contrast to longer pulses, the interaction of a 7 fs (2.6 optical cycles) pulse and fused silica is characterized by high ionization levels at low fluences and low energy deposition at high fluences. As the fluence increases, the electron density rises steeply and approaches $n_{0}/4$ at a low fluence near 1.9 J/cm^2^, compared to 2.6 J/cm^2^ and 3.7 J/cm^2^ for 25 fs and 100 fs pulses, respectively. These dense plasmas screen the incident light so efficiently that less energy is deposited into the target (see Figure 4b–d).

For the cases of 25 fs and 100 fs pulses, transmission is near 30% even when the electron density has reached $n_{0}/4$ at the surface, which is consistent with Ref. [54]. This high transmission is mostly attributed to the rising edge of the pulse before the formation of dense plasma, which reveals the exaggeration of energy absorption in the steady-state simulation with a preexisting thin, highly collisional plasma layer on the surface. However, the electron density induced by the 100 fs pulse in Figure 4a is much lower than its counterpart in Ref. [54], because Ref. [54] only applies multi-photon ionization and neglects the effect of ponderomotive energy on the bandgap in both the ionization processes, which can exaggerate the electron density.

**Figure 5:**
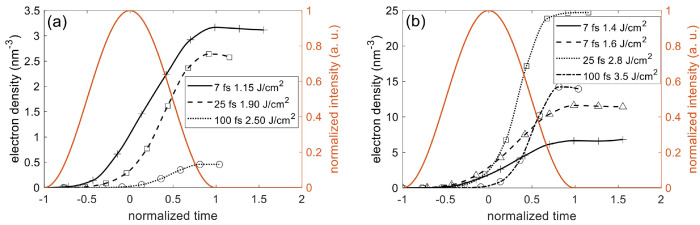
Electron density evolution for different pulse durations at LID thresholds (**a**) and ablation thresholds (**b**) based on measurements in [27,47]. The orange curve represents the intensity profile of the laser pulse and the time is normalized by the pulse duration. The crosses, squares, and circles represent the simulation data points for the 7 fs, 25 fs, and 100 fs pulses, respectively. The triangles also represent the simulation data points for the 7 fs pulse. The lines through the simulation points are merely to guide the eye.

The difference between LID induced by few cycle pulses (FCPs) and longer pulses is also revealed in Figure 5. The electron density caused by a 7 fs pulse at the LID threshold is higher than those caused by 25 fs and 100 fs pulses throughout the pulse duration. In particular, the electron density reaches 1.0 × 10 ${}^{21}$ cm^−3^ when the peak of the pulse reaches the target surface, implying that the skin depth swiftly shortens after the leading edge of the pulse due to the rapid plasma generation [41], so the pulse penetration depth and the energy absorption efficiency are limited. Consequently, the ablation crater caused by FCPs is shallower than those caused by longer pulses, which agrees with the measurements [47]. On the contrary, the electron density induced by the 100 fs pulse grows slowly and eventually reaches a lower value at the LID threshold than those caused by the shorter pulses. However, such low electron density is sufficient to yield damage in this case. The long skin depth makes the electron heating more efficient, and the interaction of pulse and solid lasts much longer, so a considerable amount of energy can be deposited into the solid.

Figure 4a and Figure 5b show that the electron density exceeds the critical density by a factor of four at the ablation thresholds for all pulse durations. Such high ionization level implies considerable broken covalent bonds. Under this circumstance, ions in an amorphous dielectric target are more loosely bonded than those in metallic crystals. Atoms in metals are arranged like closely-packed spheres due to the electrostatic interactions between mobile electrons surround a positive nuclei. The high absorbed energy density will overcome these loose bonds and lead to ablation. As shown in Figure 5b, although the 25 fs and 100 fs pulses all result in electron densities over 1.0 × 10${}^{22}$ cm^−3^ at the ablation thresholds, for the case of the 7 fs pulse, it rises only up to 6.8 × 10${}^{21}$ cm^−3^. It is possible that the ablation threshold in Ref. [47] is relatively low (possibly due to sample quality variation), since the ablation threshold is 1.6 J/cm^2^ for a 5 fs pulse at λ = 780 nm in Ref. [27], and the electron density obtained from the simulation exceeds 1.0 × 10${}^{22}$ cm^−3^ at 1.6 J/cm^2^ in Figure 5b. We also note that estimations of the Keldysh parameter [32] obtained from the peak intensities at LID and ablation thresholds deliver values of about 0.5. Therefore, the tunneling regime of the photoionization dominates under those conditions of laser-material interactions. Based on the definition of the Keldysh parameter, we estimate the effective tunneling time as 1.39 fs at laser wavelength of 800 nm and peak intensity of 100 TW/cm^2^ that is shorter than the minimum electron-particle collision time attained at the simulated magnitudes of free-electron density. Therefore, the simulation results suggest the tunneling is not significantly perturbed by the collisions in the first approximation.

While there is no consensus on the most accurate LID threshold criterion, the critical density criterion is most commonly used. It has yielded results consistent with measurements for ∼100 fs pulses at near IR wavelengths, but it is not valid in general for longer wavelengths [66]. An alternative criterion is the dissociation energy of fused silica, $U_{b}=$ 46–68 kJ/cm^−3^ [54,86,87]. The melting temperature criterion has also been proposed, in which the target temperature is calculated by the two temperature model (TTM) in a longer time scale. However, the TTM involves fitting parameters (e.g., the electron-lattice coupling parameter) not strongly supported by any measurements [47].

**Figure 6:**
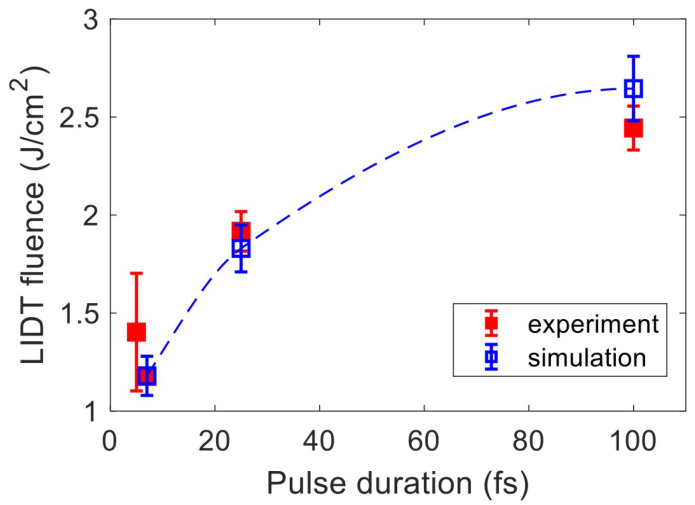
Laser damage fluence thresholds as function of laser pulse duration: simulation results compared to experimental data [27,47]. The dashed blue line through the simulation points is merely to guide the eye.

In this paper, we apply the energetic criterion to precisely benchmark our model. Assuming that most absorbed energy is deposited within a depth commensurate with the crater depth caused by pulses at the ablation threshold, the absorbed energy density can be estimated by dividing the cumulative absorbed fluence by the crater volume. The atomic force microscopic measurements in Ref. [47] show that the crater depth is 50, 70, and 80 nm at the ablation threshold for 7 fs, 25 fs, and 100 fs pulses, respectively. The crater profiles are then used to estimate crater volumes. Using the calculated cumulative absorbed fluence in Figure 4b, we then calculated the absorbed energy density $U_{a}$ and evaluated the LID thresholds by comparing $U_{a}$ with $U_{b}$. Figure 6 shows that the simulation results are in excellent agreement with the reported experimental measurements. The deviation between them slightly increases as the pulse duration increases, but the simulation and experimental tolerance ranges still overlap with each other for all the pulse durations considered here.

According to Figure 5a, the peak density is 3.2 × 10${}^{21}$ cm^−3^, 2.1 × 10${}^{21}$ cm^−3^ and 1.0 × 10${}^{21}$ cm^−3^ for 7 fs (1.15 J/cm^2^), 25 fs (1.90 J/cm^2^) and 100 fs pulses (2.70 J/cm^2^) at the experimental LID thresholds, respectively. Compared to the critical density (1.7 × 10${}^{21}$ cm^−3^), it seems to be practical to apply the critical density criteria for pulse durations within 25–100 fs for engineering purposes. For MLD coatings and gratings, extraction of the absorbed energy density is challenging because major plasma formation may not happen at the surface. PIC algorithm can calculate the local energy absorption more precisely [79], but it is much more computationally expensive. In these cases, as the critical density criterion appears appropriate, we applied it to evaluate the LID thresholds of the MLD mirror and grating for 50 fs pulses in Section 3.2 and Section 3.3. The benchmarking results in Figure 5a also provide an approach to determining reasonable electron density thresholds to estimate the LID threshold of different materials for FCPs.

### 3.2. MLD Mirror

In this simulation, the material and pulse parameters are introduced in Section 2.1 and Section 2.2, with a laser pulse duration of 50 fs and a center wavelength of 800 nm. The simulation model is depicted in Figure 1b, where the pulse emerges from the left at normal incidence. The focal spot radius of the laser pulse is *R* = 12.5 μm, which is maximized to be in the proximity to a typical experimental value while maintaining computational constraints. A 14 × 14 nm^2^ spatial grid allowed high spatial resolution representation of the MLD layers (thickness of order 100 nm) with convergent simulation results while keeping the computational time reasonable.

**Figure 7:**
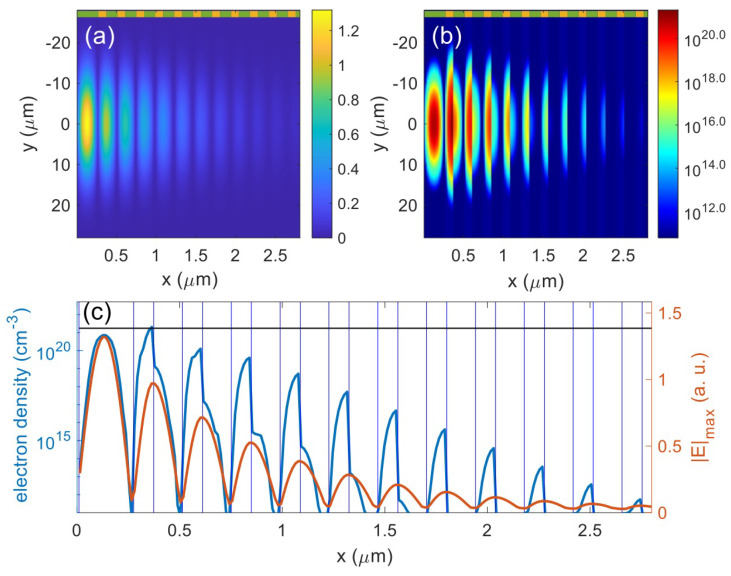
Simulation results for the MLD mirror at 0.7 J/cm^2^. (**a**) ${\left|E\right|}_{\max}$ in the mirror; (**b**) Electron density in the mirror; (**c**) Electron density and ${\left|E\right|}_{\max}$ profiles at *y* = 0. The green (SiO_2_) and yellow (HfO_2_) blocks indicate different materials. High electron density is only seen in the top layers, so only the top layers are shown here. The peak of the pulse impinges the mirror surface at *y* = 0 and *x* = 0 from the left. The black horizontal line denotes the critical density.

${\left|E\right|}_{\max}$ is the maximum electric field strength at each cell during the simulation, and it is normalized to the amplitude of the incident pulse. Compared to the electric field enhancement calculated by spectrum-based approaches, ${\left|E\right|}_{\max}$ reflects the local field enhancement caused by ultra-short laser pulses. Figure 7 suggests that the electron density distribution is strongly correlated to ${\left|E\right|}_{\max}$, but such correlation varies among different materials. As shown in Figure 7c, ${\left|E\right|}_{\max}$ is continuous along the depth but the electron density changes greatly at the interfaces. The peak of ${\left|E\right|}_{\max}$ occurs in the top SiO ${}_{2}$ layer, while the peak electron density is in the second layer made of HfO_2_. The peak electron density in the second layer made of HfO_2_ is 150 times that in the layer beneath, but ${\left|E\right|}_{\max}$ does not vary at the intermediate interface. This outstanding different electron density is mainly caused by the different bandgaps of SiO_2_ and HfO_2_. The critical electron density criterion ($n_{\mathrm{cr}}\approx 1.74\times 10^{21}$ cm^−3^) suggests that the LID threshold fluence is 0.67 J/cm^2^. The interface beneath the second layer (HfO_2_) is most likely damaged at the LID threshold, and the top SiO_2_ layer will be probably damaged in the middle at a slightly higher fluence. This result implies that if one can gradually increase the incident fluence, the damage morphology will present a progression of blistering, ablation, ablation upon blistering [31], and so forth, and progress deeper into the stack.

### 3.3. MLD Grating

**Figure 8:**
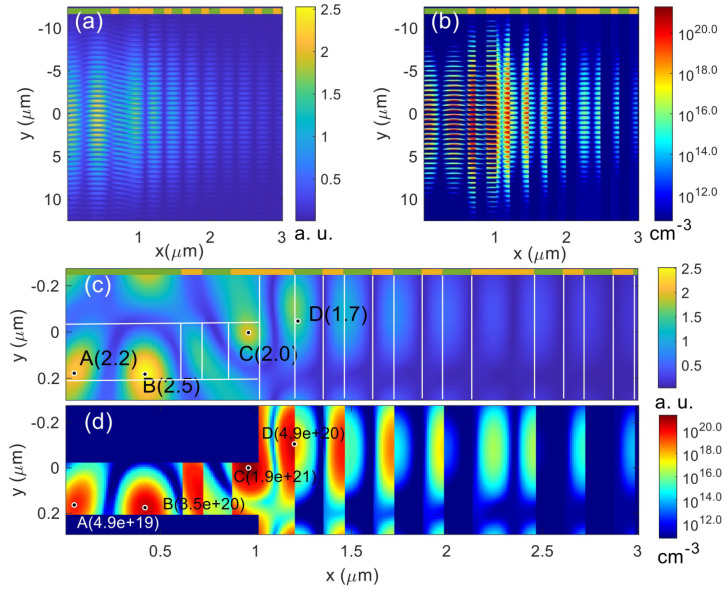
Simulation results of a MLD grating at 0.18 J/cm^2^. (**a**) ${\left|E\right|}_{\max}$ in the grating; (**b**) Electron density in the grating; (**c**,**d**) are the local distributions of ${\left|E\right|}_{\max}$ and electron density profiles near *y* = 0, respectively. The green (SiO_2_) and yellow (HfO_2_) indicate the materials in each layers. The high electron density is only seen in the top layers near the LID threshold, so the Ta_2_O_5_ layers near the substrate are not shown. The *s*-polarized pulse center impinges on the grating surface at *y* = 0 and *x* = 0 from the left at AOI = 38°.

In this simulation, a 50 fs pulse (*R* = 5.3 μm) is incident upon the MLD grating from the upper left at AOI = 38° (see Figure 1c). The material and pulse parameters are introduced in Section 2.1. Compared to the simulation results of the MLD mirror, ${\left|E\right|}_{\max}$ in the MLD grating is much higher (see Figure 8a), especially at the grating pillars and top layers in the thin-film stack. Therefore, the critical density is achieved at a much lower fluence, 0.18 J/cm^2^, and the LID threshold of the MLD grating is estimated as 0.17 J/cm^2^. A 10 × 10 nm^2^ spatial grid is used here to obtain convergent simulation results.

Similarly, the electron density distribution is correlated with ${\left|E\right|}_{\max}$, but HfO_2_ is more rapidly ionized than SiO_2_. As shown in Figure 8c,d, the maximum ${\left|E\right|}_{\max}$ occurs at the top of pillars made of SiO_2_ (see point B in Figure 8c), while the maximum electron density locates in the bottom of pillars made of HfO_2_ (see point C in Figure 8d). The second peak of electron density is at the interface between the first HfO_2_ and SiO_2_ layers (see point D in Figure 8d). Therefore, the simulation suggests that, at the LID threshold, damage is initiated most likely at the bottom of pillars at the side facing the incident pulse. At slightly higher fluences, damage at the bottom and top of pillars, and deformation at the first HfO_2_/SiO_2_ interface can occur at the same time. Since the grating pillars and the top layers are subjected to the highest field enhancement, high-bandgap dielectrics are better candidates for the grating pillars and the top layers where the field enhancement is highest.

## 4. Discussion

### 4.1. Modified Keldysh Formula

The widely applied Keldysh formula for the non-parabolic band structure in Ref. [32] has a small misprint (see Supplementary Materials). The modified/correct Keldysh formulae are expressed as [51]

(16) $$\omega_{\mathrm{ph}}=2\frac{2\omega }{9\pi }{\left(\frac{\omega m^{*}}{\hbar \gamma_{1}}\right)}^{\frac{3}{2}}Q\left(\gamma ,x\right)\times \exp\left[-\pi \left\lfloor x+1\right\rfloor \frac{K\left(\gamma_{1}\right)-E\left(\gamma_{1}\right)}{E(\gamma_{2})}\right],$$

(17) $$Q\left(\gamma ,x\right)=\sqrt{\frac{\pi }{2K(\gamma_{2})}}\sum_{n=0}^{\infty }\exp\left[-\pi n\frac{K\left(\gamma_{1}\right)-E\left(\gamma_{1}\right)}{E(\gamma_{2})}\right]\times \Phi \left[\pi \sqrt{\frac{\lfloor x+1\rfloor -x+n}{2K\left(\gamma_{2}\right)E\left(\gamma_{2}\right)}}\right],$$

where *K* and *E* are the complete elliptic integrals of the first and second kind; Φ is the Dawson function; $\gamma_{1}=\gamma /\sqrt{\left(1+\gamma^{2}\right)}$; $\gamma_{2}=1/\sqrt{\left(1+\gamma^{2}\right)}$. The Keldysh parameter $\gamma =\omega \sqrt{m^{*}\Delta }/\left(eA\right)$, where *A* is the amplitude of the laser pulse. $x=\epsilon_{\mathrm{eff}}/\left(\hbar \omega \right)$, where $\epsilon_{\mathrm{eff}}=2\Delta E\left(\gamma_{2}\right)/\left(\pi \gamma_{1}\right)$, and Δ is the intrinsic bandgap.

**Figure 9:**
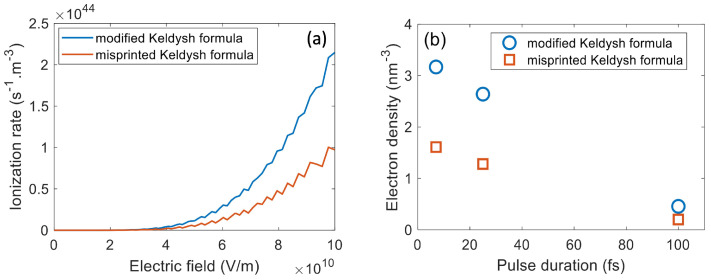
Comparison of (**a**) photoionization rates and (**b**) electron densities at LID threshold calculated by the uncorrected and corrected/modified Keldysh formulae for fused silica. The incident fluence is 1.15, 1.90, and 2.50 J/cm^2^ for pulse duration of 7, 25, and 100 fs, respectively. λ = 800 nm. The pulses are *s*-polarized.

**Figure 10:**
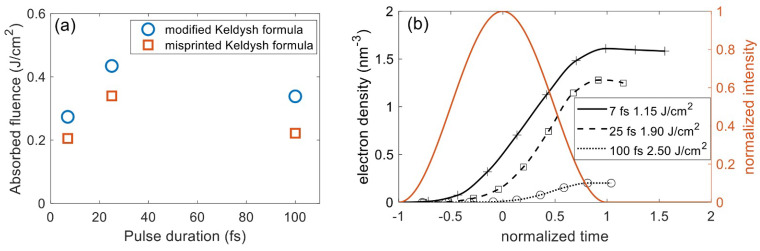
Comparison of the cumulative absorbed fluence (**a**) and electron density evolution (**b**) at LID threshold fluence calculated by the uncorrected and modified/corrected Keldysh formulae. The orange curve represents the intensity profile of the laser pulse and the time is normalized by the pulse duration. The pulse parameters are the same as those in Figure 9. The crosses, squares, and circles in (**b**) represent the simulation data points for the 7 fs, 25 fs, and 100 fs pulses, respectively.

Figure 9 shows that the photoionization rate and electron density obtained from the unmodified equations are almost half of those obtained from Equations (16) and (17). Especially, by using the uncorrected formulae, the electron density is lower than the critical density even for all pulse durations at the LID thresholds. Figure 10a shows that the misprinted equation results in a lower cumulative absorbed fluence, and this impact increases as the pulse duration becomes longer. The electron density during the interaction in Figure 10b, which is calculated from unmodified Equations (16) and (17), is much lower than the corrected result in Figure 5a.

These deviations can impact simulation results substantially. Unfortunately, many previous works applied the misprinted equations in calculation of the photoionization rate [46,47,48,49,50]. However, some papers based on the uncorrected formulae still report electron densities several times higher than the critical density at LID threshold fluences. Extra compensation may contribute to such high electron density, such as using a non-Keldysh impact ionization model that exaggerates the avalanche [88], or using the electric field amplitude not reduced by the polarization in dielectrics to calculate the Keldysh parameter γ [47].

### 4.2. Impact Ionization Energy Threshold

**Figure 11:**
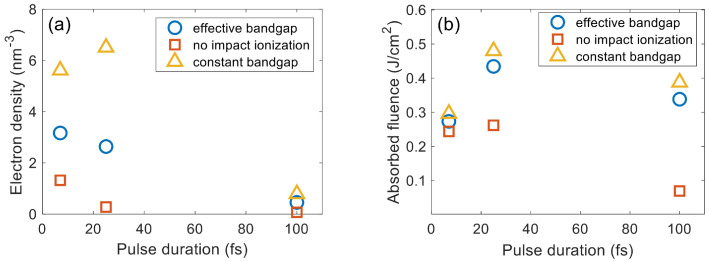
Comparison of (**a**) electron density and (**b**) absorbed fluence calculated from the simulation without impact ionization and the simulations with ionization but using effective and constant bandgaps. The pulse parameters are the same as those in Figure 9.

Many studies consider the ponderomotive energy for the evaluation of effective band gap based on the Keldysh formula but ignore its effect on impact ionization [88,89,90,91]. For example, in the case of fused silica, the effective bandgap can be 10% to 50% higher for the intensities 10${}^{13}$ Wcm^−2^ and 10${}^{14}$ Wcm^−2^ in the near-IR wavelengths, respectively. Based on the impact ionization model presented in Section 2.2.2, we compared the results obtained for fused silica with the intrinsic bandgap and the effective bandgap. Figure 11 shows that utilizing the intrinsic bandgap will overestimate the electron density by over a factor of two. The absorbed fluence does not increase much for the 7 fs pulse, but this deviation rises as the pulse duration increases. Therefore, the failure to consider the bandgap shift will greatly underestimate the LID threshold fluence if one applies the critical electron density criterion, but the underestimation is milder using the energetic criterion. Meanwhile, the absence of the impact ionization can overestimate the LID threshold based on both criteria.

### 4.3. Electric Field Amplitude for Keldysh Photoionization Formulae

**Figure 12:**
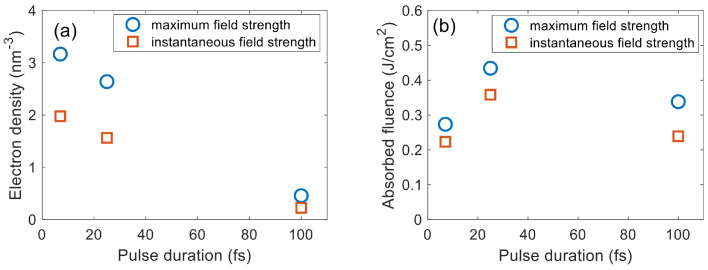
Comparison of (**a**) electron density and (**b**) absorbed fluence in fused silica calculated from the Keldysh photoionization rates using the instantaneous electric field strength vs. the maximum field strength in an optical cycle, respectively. The pulse parameters are the same as those in Figure 9.

As mentioned beforehand, the Keldysh formula assumes a slowly varying amplitude of the pulse in an optical cycle, which is incompatible with dynamic schemes that calculate the instantaneous field strength. In order to minimize the incompatibility, we approximate the maximum electric field strength in an optical cycle as the amplitude in this specific cycle. As shown in Figure 12, using the instantaneous field strength will underestimate the electron density and energy deposition significantly.

## 5. Conclusions

In summary, we have proposed a 2D dynamic coupled model to simulate the interaction of femtosecond laser pulses and dielectrics. The model is benchmarked using bulk fused silica, and the simulation results agree well with the experimental results based on the energetic LID threshold criterion. A comprehensive theoretical analysis of the strong field electronic excitation and pulse energy deposition has been performed for different pulse durations and incident fluences. Higher plasma density and less energy deposition are observed in the simulation results for FCPs compared to longer pulses. For pulse duration from 25 fs to 100 fs, the simulation results suggest that the electron density criterion can be applied for the ease of practical use, especially for those who are seeking to engineer and predict the LID threshold of MLD mirrors and gratings. In this paper, we estimated the LID threshold fluences of a MLD mirror and a MLD grating, which are 0.67 J/cm^2^ and 0.17 J/cm^2^, respectively. The simulation results also suggest the progression of the damage morphology versus the incident fluences.

Several crucial aspects in implementing the Keldysh photoionization theory and impact ionization model for pulse duration < 100 fs have been discussed and analyzed, including the misprint in Ref. [32], an approach to estimate the slowly-varying electric-field amplitude in the Keldysh formula, and the effect of ponderomotive energy on the impact ionization rate. Quantitative comparisons for fused silica show that these aspects can make a significant difference to the simulation results. The plasma density and energy deposition will be underestimated if one adopts the misprint or calculates the Keldysh parameter using the instantaneous electric field strength. Meanwhile, the plasma density and energy deposition will be exaggerated if the ponderomotive energy is ignored in the impact ionization model. The authors hope the improvements of the simulation approach outlined in this paper will benefit a broad range of researchers in the fields of modeling ultrafast high-intensity laser-solid interactions.

The model presented here is by no means complete, and several upgrades are currently being considered. Among them, we should mention the two that stand out. First is the need of proper implementation of the few-cycle photoionization model [78]. This work can potentially result in the development of a novel type of dynamic simulations for the few-cycle laser propagation coupled to highly non-equilibrium time-dependent material response. Second, there is a real need to incorporate the non-thermal and ballistic nature of the laser accelerated free carriers in the ultrashort pulse laser damage model, as it affects many aspects of the damage process, including impact and avalanche ionization. Efforts are under way in both these fronts, whose results will be presented in the near future.

## Figures and Tables

**Table 1:** Material parameters for simulation [57].

Materials	$m^{*}$	Bandgap	*n*	Mass Density	$\tau_{\mathrm{recomb}}$
SiO_2_	0.56 $m_{e}$	9 eV	1.45	2.20 g/cc	220 fs
HfO_2_	0.55 $m_{e}$	5.7 eV	1.98	9.68 g/cc	1050 fs
Ta_2_O_5_	0.61 $m_{e}$	4.3 eV	2.07	8.20 g/cc	490 fs

**Table 2:** Nomenclature of derived variables in FDTD algorithm.

Variables	Definition	Variables	Definition
$T_{e}$ [eV]	electron temperature	$\epsilon_{\mathrm{eff}}$ [eV]	effective bandgap
$n_{e}$ [m^−3^]	CB electron density	$J_{D}$ [A·m^−2^]	plasma current
$\omega_{\mathrm{ph}}$ [s^−1^]	Keldysh ionization rate	$\omega_{\mathrm{col}}$ [s^−1^]	impact ionization rate
*E* [V/m]	electric field	β [s^−1^]	electron collision frequency
*B* [T]	magnetic field	$J_{p}$ [A·m^−2^]	current attributed to photoionization

## Data Availability

Simulation raw data may be made available upon reasonable request to the authors.

## Supplementary Materials

The Keldysh ionization rate formula with the misprint error is available online at https://www.mdpi.com/article/10.3390/nano12081259/s1.
