# Supplementary material for: Ultrafast Laser Material Damage Simulation—A New Look at an Old Problem
Source: Nanomaterials (Basel). 2022 Apr 8;12(8):1259. doi: 10.3390/nano12081259 (PMC9031137; doi:10.3390/nano12081259)
Supplement: Supplementary file 1 [file nanomaterials-12-01259-s001.zip › nanomaterials-1515794-supplementary.pdf]

Supplementary Material

Ultrafast Laser Material Damage Simulation – A New Look at an Old Problem

Simin Zhang 1 , Carmen Menoni 2,3 , Vitaly Gruzdev 4 and Enam Chowdhury1,5,6*

1 Department of Material Science and Engineering, The Ohio State University, Columbus, OH 43210, USA;
zhang.10584@osu.edu2 Electrical and Computer Engineering Department, Colorado State University, Fort Collins, CO 80523;
carmen.menoni@colostate.edu3 XUV Lasers Inc., Fort Collins, CO 80525, USA;4 Department of Mechanical and Aerospace Engineering, University of Missouri, Columbia, MO 65211,
USA; vgruzdev@unm.edu5 Department of Physics, The Ohio State University, Columbus, OH 43210, USA;6 Department of Electrical and Computer Engineering, The Ohio State University, Columbus, OH 43210,
USA

* Correspondence: chowdhury.24@osu.edu

The widely applied Keldysh formula is for the non-parabolic band structure has a small misprint compared to Eqs. 16 and 17, which is expressed in Ref.[32] as

$$\omega_{\mathrm{ph}} = \frac{2\omega}{9\pi}\left(\frac{\omega m^*}{\hbar\gamma_1}\right)^{\frac{3}{2}} Q(\gamma, x) \times \exp\left[-\pi\lfloor x+1\rfloor \frac{K(\gamma_1)-E(\gamma_1)}{E(\gamma_2)}\right], \quad \text{(S1)}$$

$$Q(\gamma, x) = \sqrt{\frac{\pi}{2K(\gamma_2)}}\sum_{n=0}^{\infty} \exp\left[-\pi n \frac{K(\gamma_1)-E(\gamma_1)}{E(\gamma_2)}\right] \times \Phi\left[\pi\sqrt{\frac{2\lfloor x+1\rfloor-2x+n}{2K(\gamma_2)E(\gamma_2)}}\right], \quad \text{(S2)}$$

where K and E are the complete elliptic integrals of the first and second kind; Φ is the Dawson function; $\gamma_1 = \gamma/\sqrt{(1+\gamma^2)}$; $\gamma_2 = 1/\sqrt{(1+\gamma^2)}$. The Keldysh parameter $\gamma = \omega\sqrt{m^*\Delta}/(eA)$, where A is the amplitude of the laser pulse. $x = \epsilon_{\mathrm{eff}}/(\hbar\omega)$, where $\epsilon_{\mathrm{eff}} = 2\Delta E(\gamma_2)/(\pi\gamma_1)$, and Δ is the intrinsic bandgap.
